# Quality by design approach for development and validation of a RP-HPLC method for simultaneous estimation of xipamide and valsartan in human plasma

**DOI:** 10.1186/s13065-022-00864-4

**Published:** 2022-09-20

**Authors:** Mahmoud M. Sebaiy, Sobhy M. El-Adl, Mohamed M. Baraka, Amira A. Hassan, Heba M. El-Sayed

**Affiliations:** 1 Medicinal Chemistry Department, Faculty of Pharmacy, Zagazig University, Zagazig, Egypt; 2 Analytical Chemistry Department, Faculty of Pharmacy, Zagazig University, Zagazig, Egypt

**Keywords:** RP-HPLC, QbD, Xipamide, Valsartan, Human plasma

## Abstract

A new rapid, simple, and sensitive RP-HPLC method was carried out through applying Quality by Design approach for determination of xipamide and valsartan in Human plasma. Fractional factorial design was used for screening of four independent factors: pH, flow rate, detection wavelength, and % of MeOH. Analysis of variance (ANOVA) confirmed that flow rate and % of MeOH were only significant. Chromatographic conditions optimization was carried out through using central composite design. Method analysis was performed using BDS Hypersil C8 column (250 × 4.6 mm, 5 μm) and an isocratic mobile phase of MeOH and 0.05 M KH_2_PO_4_ buffer pH 3 (64.5:35.5, v/v) at 1.2 mL/min flow rate with UV detection at 240 nm and 10 μL injection volume. According to FDA guidelines, the method was then validated for the determination of the two drugs clinically in human plasma in respect of future pharmacokinetic and bioequivalence simulation studies. The standard curve was linear in the concentration range of 5–100 µg/mL for both drugs, with a determination coefficient (R^2^) of 0.999. Also, the average recoveries lied within the range from 99.89 to 100.03%. The proposed method showed good predictability and robustness.

## Introduction

Quality by design (QbD) is a modern and systematic approach for quality control of pharmaceuticals and product development. Pharmaceutical quality can be assured by understanding and controlling variable parameters for formulation and manufacturing processes through such structured context [1–3]. Now-a-days the concept of QbD can be extended to analytical and bioanalytical techniques. The application of QbD principles can help in clinical laboratories to develop a suitable analytical method providing a significant improvement better than the traditional and empirical methodology [4]. One of these QbD approaches is fractional factorial design (FFD) which is commonly used and effective tool in scientific research and industrial applications. The main advantage of FFD is that it allows building statistical models with a few numbers of runs. Using the models allows identification of the significant factors affecting certain responses during analytical method development. Central composite design (CCD) is an efficient tool in optimization of significant factors. CCD suggests the optimal variables value that gives the best and most desired response and defines process conditions which are robust to deliberate variations in factor settings. Also, it suggests a mathematical model relating the response with the critical variables, thus allowing to predict response with minimal error transmitted to that response (propagation of error or POE) [5].

Different classes are indicated for management of hypertension with concomitant disease. These classes include diuretics, beta-blockers, angiotensin converting enzyme (ACE) inhibitors, angiotensin receptor blockers (ARBs), and Aldosterone receptor antagonists. Diuretics have an initial decreasing effect on blood volume and consequently reduce blood pressure. ARBs have more complete blockade of angiotensin II actions compared with ACE inhibitors, so they are a substitute for the latter in treating patients with heart failure and noticeable ACE inhibitors side effects. Therefore, diuretics and ARBs can be considered as a rational drug combination for patients with hypertension associated with heart failure (HF). This combination is more effective than monotherapy with one of its components. It offers a remarkable reduction in blood pressure with lower doses and minimized adverse effects [6].

**Fig. 1:**
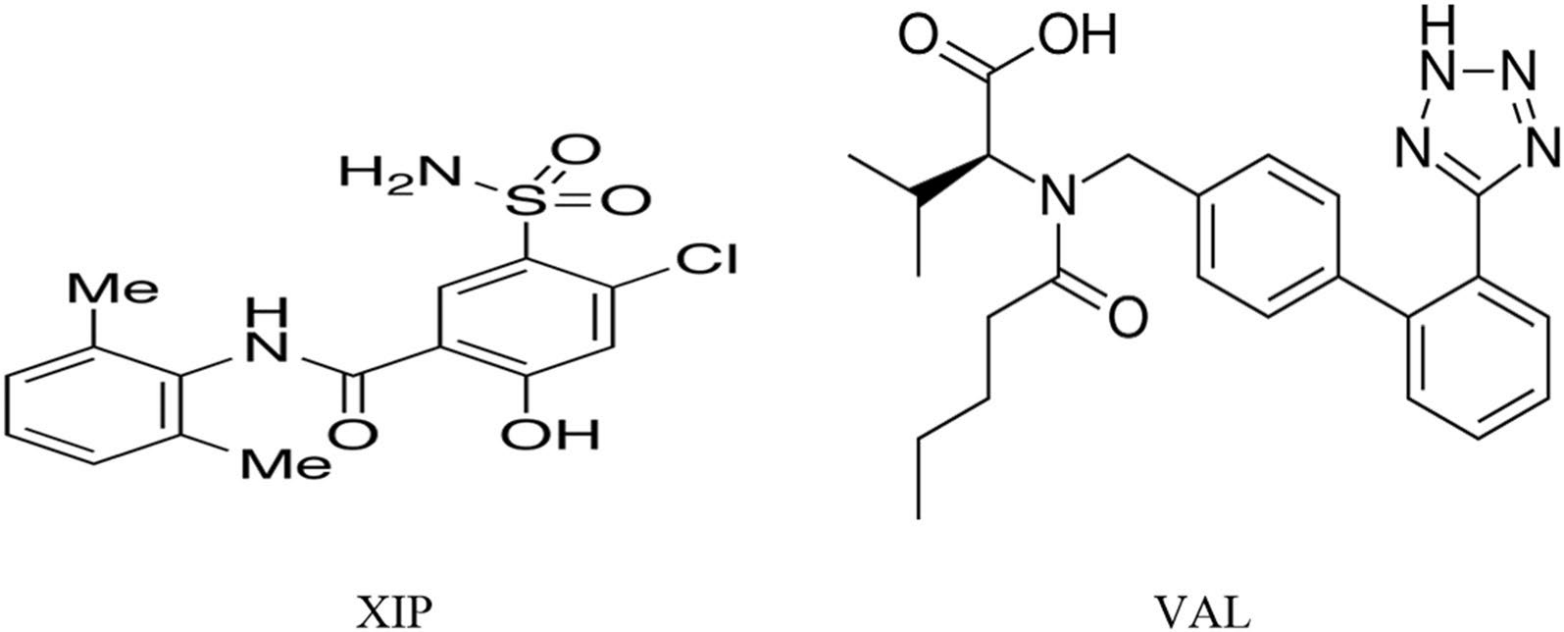
Structure of xipamide (XIP) and valsartan (VAL)

Xipamide (XIP) is a sulphonamide diuretic drug used in the treatment of hypertension either alone or in combination with other antihypertensives. It is also used in treatment of oedema including that related to HF [7]. Chemical structure of XIP, 5-(Arninosulphonyl) 4-chloro-*N*-(2,6-dimethylphenyl)-2-hydroxy-benzamide, is presented in Fig. 1. XIP acts mainly on both kidneys to reduce reabsorption of sodium in the distal convoluted tubule. The determination of XIP has been performed by HPLC [8–10], spectrophotometry [11, 12], spectroflourimetry [13], and voltammetry [14].

Valsartan (VAL) is an orally active and potent, non-peptide tetrazole derivative where it selectively inhibits Angiotensin II Receptor type 1 leading to reduction in blood pressure and so it can be used in hypertension treatment, to reduce mortality in patients with left ventricular dysfunction following myocardial infarction, and in HF management [7, 15] Chemically, it is 2(S)-3-Methyl-2-(pentanoyl{[2ʹ-(1*H*-tetrazol-5-yl)-4-biphenyl]methyl}amino) butanoic acid (Fig. 1). Literature review revealed that the determination of VAL has been carried out using HPLC [16–28], spectrophotometry [29–33] and spectroflourimetry [34, 35].

To the best of our comprehensive survey, XIP and VAL were not determined before as combined mixture (despite their synergistic action) by chromatographic techniques neither in biological nor pharmaceutical samples. As such, in line with keeping in mind the current FDA requirements while pursuing the study considering QbD based approach, the objective of our research is to develop a novel, accurate, robust, simple and specific HPLC method suitable for determination of XIP and VAL using FFD regarding pharmacokinetic and bioequivalence simulation studies and robustness testing. Among the different experimental designs, FFD as a response surface was preferrable for nonlinear response prediction in addition to its flexibility, in respect of experimental runs and information correlated with main and interaction factor effects.

## Experimental

### Apparatus

- Agilent 1200^®^ HPLC instrument (Germany) with a Thermo Scientific^®^ BDS Hypersil C_8_ column (5 µm, 250 × 4.60 mm), DAD absorbance detector, in addition to HPLC QUAT pumps are connected to PC computer which is loaded with Agilent 1200 software [36, 37].
- Labomed^®^ Spectro (U6VD-2950) UV–VIS Double Beam Spectrophotometer (England) with 1 cm quartz cells and connected to PC computer loaded with UVWin5 Software v6 [36, 37].
- HANNA^®^ HI 8314 (Romania) membrane pH-meter for pH adjustment [37].

### Materials and Reagents

- All materials, chemicals, and solvents were of HPLC grade [37].
- XIP (99.79%) and VAL (99.90%) were obtained from EIPICO (Tenth of Ramadan City, Egypt). Standard solutions of 200 µg/mL were prepared through dissolving 10 mg of each pure drug in 50 mL of the mobile phase [36].
- Mobile phase was a binary mixture (freshly prepared) of MEOH: 0.05 M potassium dihydrogen phosphate (64.5: 35.5, v/v) adjusted to pH 3 by using ortho-phosphoric acid, filtered and degassed by using 0.45 µm membrane filters (Millipore, USA) [36].
- MeOH (Fischer Scientific, Hampton, USA), Potassium dihydrogen phosphate (Techno Pharmchem, Delhi, India) and orthophosphoric acid (Merck, India) were all analytical grade assigned [33].
- The human plasma was provided kindly by Zagazig University Hospital and was labeled to be disease and drug free. It was kept frozen at −20 °C before initial use and was then stored at −4 °C during usual uses [37].

### Procedures

#### Construction of calibration curves

Appropriate mixed dilutions of XIP and VAL standard stock solutions were done in 10 mL volumetric flasks to get final concentrations of 5, 12.5, 25, 50 and 100 µg/mL for both drugs. A 10 μL of each mixture was injected then into the column while the chromatogram was monitored at 240 nm. A calibration graph was plotted as drug concentration against peak area response [37].

#### Human plasma samples procedure

All experimental protocols in the current study were approved by the EGYPTIAN NETWORK OF RESEARCH ETHICS COMMITTEES at the Faculty of Pharmacy, Zagazig University (Approved 2008). Calibration curves and validation QC samples in plasma at various concentrations of 2.50, 5, 15 and 20 µg/mL were prepared. Aliquots of 200 µL plasma samples and various drug mixture volumes ranging from 100–200 µL were added to 10 mL centrifuge tubes and then vortexed for 1 min. After that, the mixture was precipitated using methanol (total volume is 2 mL). After vortexing for 1 min, the samples were then centrifuged at 5000 rpm for 15 min. Aliquots of 10 µL of each supernatant was filtered using 0.45 µm PTFE syringe filters (Membrane solutions, USA) and directly injected into HPLC instrument for analysis [37].

### Experimental design

#### Scouting step

Some trials were included in this step to find out a suitable mobile phase that can give an acceptable separation for both drugs. At the beginning, different concentrations containing either 0.025 or 0.05 M KH_2_PO_4_ buffer (as an aqueous part of the mobile phase) were tried. In addition, acetonitrile and MeOH were tested as organic modifiers. Finally, the variables that may clearly affect the selected responses were chosen [38].

#### Screening design

A resolution IV FFD with a minimum number of runs was used to identify the significant factors affecting the measured responses (Table 1). In this study, 4 independent factors were tested at 2 levels; pH at 3 & 4, flow rate at and 1.2 mL/min, detection wavelength at 230 and 250 nm, and also % of MeOH at 58 and 63%. The mathematical model related to the design consists of main effects and possible interaction effects (2 FI). In this case, 2 responses were taken into consideration: retention time (VAL) and resolution [39].

**Table 1:** Resolution IV fractional factorial screening design for determination of XIP and VAL by RP-HPLC

Std.	Run	Factor 1	Factor 2	Factor 3	Factor 4	Factor 5	Response 1	Response 2
		A: pH	B: % MeOH	C: Flow rate (mL/min)	D: Detection wavelength (nm)	E: Buffer conc (Mm)	Resolution	Retention time (VAL) (min)
5	1	4	58	1.2	250	0.05	7.6	6.91
9	2	4	58	1	250	0.025	7.56	8
7	3	4	63	1.2	230	0.025	4.31	4.56
4	4	3	63	1.2	230	0.05	5.86	5.2
8	5	4	58	1	230	0.05	7.75	8.2
2	6	3	58	1	250	0.05	9.13	9.27
10	7	3	63	1	230	0.025	3.9	5.23
1	8	4	63	1	250	0.05	4.6	5.5
6	9	3	58	1.2	230	0.025	8.53	7.68
3	10	3	63	1.2	250	0.025	3.8	4.36

#### Optimization design

Central composite design (CCD) was commonly used due to its high efficiency and capability to reduce number of runs. A CCD with k factors should require 2 Table 2 k factorial runs, 2k axial experiments, symmetrically spaced at ± α along each variable axis, and one center point at least [40]. A rotatable CCD (α = 1.68) was built for the 4 significant factors to get the optimum level for desired responses using 5 levels of each factor (− α, − 1, 0, + 1, + α) with total number of 13 random runs which are including 5 center points (Table 2). The technique of numerical optimization and desirability function approach are used together usually to locate the optimized conditions through different trading off selected responses [41]. In this study, the numerical optimization was based on minimizing retention time (VAL) (+++ importance) and maximizing resolution (+ importance) between the analytes, obtaining a reasonable desirability function, and minimizing POE of both responses (+++ importance) to ensure that minimum error was transferred to responses.

**Table 2:** Central composite design for optimization with the measured responses

Std.	Run	Factor 1	Factor 2	Response 1	Response 2
		A: % MeOH	B: Flow rate (mL/min)	Retention time (VAL) (min)	Resolution
12	1	62.5	1.1	5.99	6.23
6	2	66.0355	1.1	4.6	4.18
4	3	65	1.2	4.57	4.77
11	4	62.5	1.1	5.87	6.2
3	5	60	1.2	6.6	7.29
1	6	60	1	8	7.81
13	7	62.5	1.1	5.79	6.11
10	8	62.5	1.1	5.79	6.16
5	9	58.9645	1.1	7.68	8.48
9	10	62.5	1.1	5.76	6.16
8	11	62.5	1.24142	5.17	6.09
2	12	65	1	5.45	4.75
7	13	62.5	0.958579	6.6	6.27

Another tool was graphical optimization used to specify the design space (sweet spot) where desired CQAs meet. Graphical optimization goal was to minimize retention time (VAL) to be less than 6 min., and maximizing resolution with 3.6 as a lower limit, as well as, to minimize a POE of both responses by adjusting the highest acceptable upper limit. In addition, interval criteria were applied for CQAs and POE to understand the uncertainty impact on achieving the process goals. The sweet spot (sometimes called the bright yellow area) was obtained for each two variables, whilst the remaining factors were kept at a certain fixed value.

Finally, model predictability confirmation was checked through assuring that the predicted means of retention time (VAL), resolution and their POE lie within the low & high 95% values of prediction interval (PI low 95% and PI high 95%).

Investigation of model predictability was also achieved through prediction error calculation in accordance with the following equation [42]:

$$ {\text{Prediction}}\,{\text{error}} = \,{{({\text{Observed}} - {\text{predicted}})} \mathord{\left/ {\vphantom {{({\text{Observed}} - {\text{predicted}})} {{\text{predicted}}}}} \right. \kern-\nulldelimiterspace} {{\text{predicted}}}} \times 100. $$

## Results and discussion

### Chromatographic conditions optimization

**Fig. 2:**
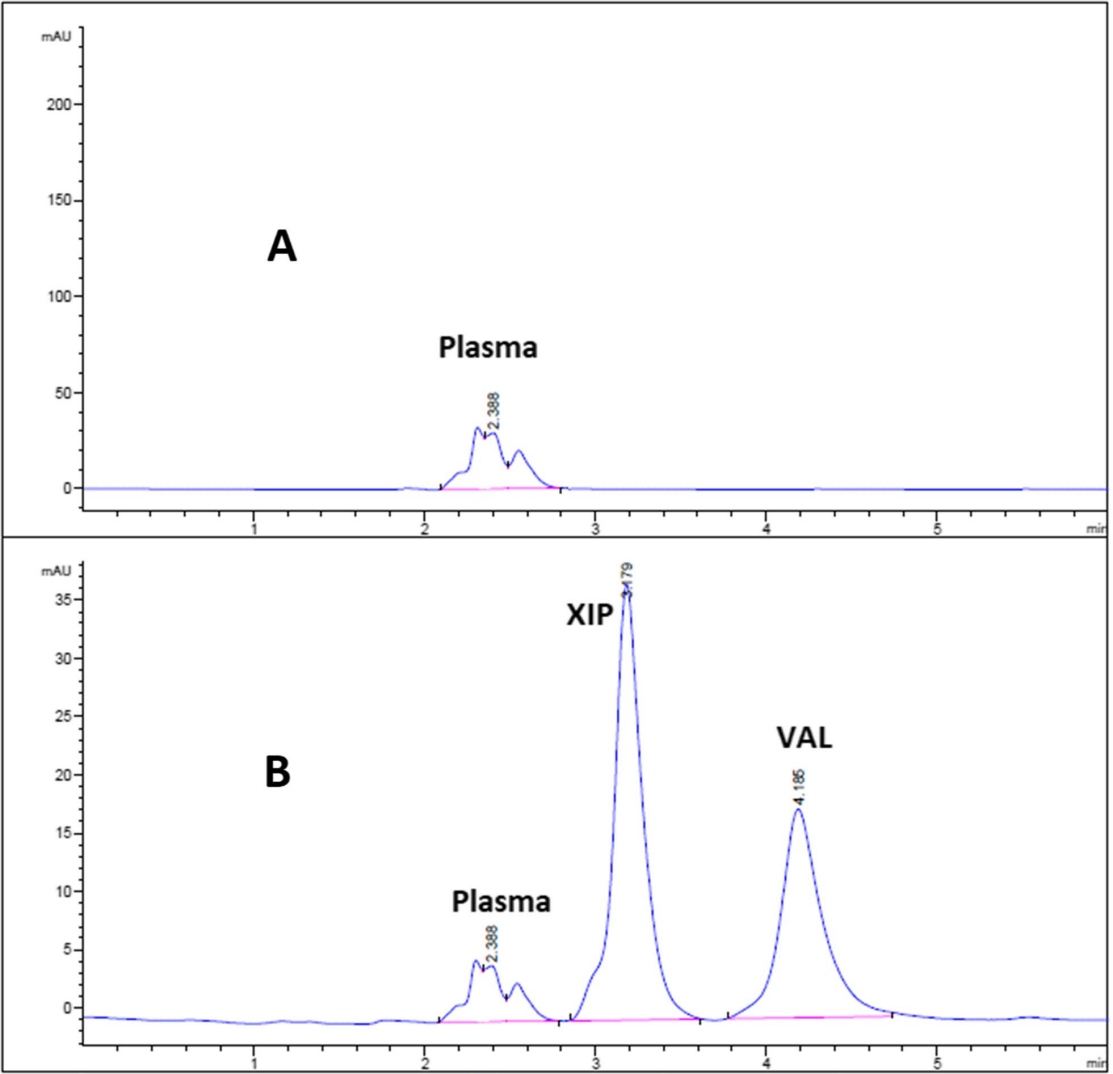
HPLC chromatogram of (**A**) blank plasma (**B**) mixture of 12.50 µg/mL XIP and VAL in human plasma sample

All chromatographic conditions are detailed in Table 3. Spectral analysis of both drugs in the range of 200–400 nm showed that XIP and VAL have λ_max_ at 237 nm and 250 nm, respectively. As such, the chromatographic detection was set at 240 nm using a DAD detector as the appropriate wavelength. The method was carried out using a Thermo Scientific^®^ BDS Hypersil C_8_ column (5 µm, 250 × 4.60 mm). The optimum mobile phase was determined as a MeOH: 0.05 M potassium dihydrogen phosphate mixture adjusted to pH 3 by using ortho-phosphoric acid (64.5: 35.5, v/v) at a flow rate of 1.2 mL/min. Under such conditions, XIP and VAL in human plasma can be completely separated at 3.23 and 4.34 min respectively as depicted in Fig. 2B, respectively. In addition, the mixture in plasma didn’t exhibit any matrix interference effect where human plasma chromatogram (Fig. 2A) showed no peaks at retention times of XIP and VAL.

**Table 3:** Chromatographic conditions for the proposed HPLC method for estimation of XIP and VAL

Parameters	Conditions
Column	Thermo Scientific^®^ BDS Hypersil C_8_ 5 µm (250 × 4.60 mm)
Mobile phase	Isocratic binary mobile phase of MeOH: 0.025 M KH_2_PO_4_ adjusted to pH 3 using ortho-phosphoric acid (64.5: 35.5, v/v), filtered and degassed using 0.45 µm membrane filter
UV detection, nm	240
Flow rate, ml/min	1.2
Injected volume, µl	10
Pressure, psig	98
Temperature	Ambient

The optimal mobile phase showed good symmetrical peaks (0.8 < T < 1.2), capacity factor (1 < k < 10), and resolution higher than 2 and theoretical plates more than 2000. Table 4 shows all system suitability parameters of the proposed HPLC method for simultaneous determination of those two drugs in pure and plasma matrices.

**Table 4:** System suitability parameters for XIP and VAL in both pure and plasma samples

Parameters	Pure sample		Plasma sample		Reference values [39]
	XIP	VAL	XIP	VAL	
Retention time, t_R_	3.35 ± SD	4.66 ± SD	3.23 ± SD	4.34 ± SD	
Capacity factor, k'	1.58	2.59	1.49	2.26	Accepted kʹ value (1–10)
Peak asymmetry (Tailing factor, T)	1.00	0.92	1.18	1.15	Accepted T value ≤ 2
Theoretical plates, N	3620	3587	3384	3554	Accepted N value > 2000
Resolution, Rs	4.91		4.59		Accepted value > 2
Selectivity (separation factor, α)	1.64		1.52		

#### Scouting step

This step explains the effect of different mobile phases on analysis of the two analytes. In this step, four factors were chosen; pH, flow rate, detection wavelength, and % of MeOH to be tested in screening step.

#### Screening with FFD

**Fig. 3:**
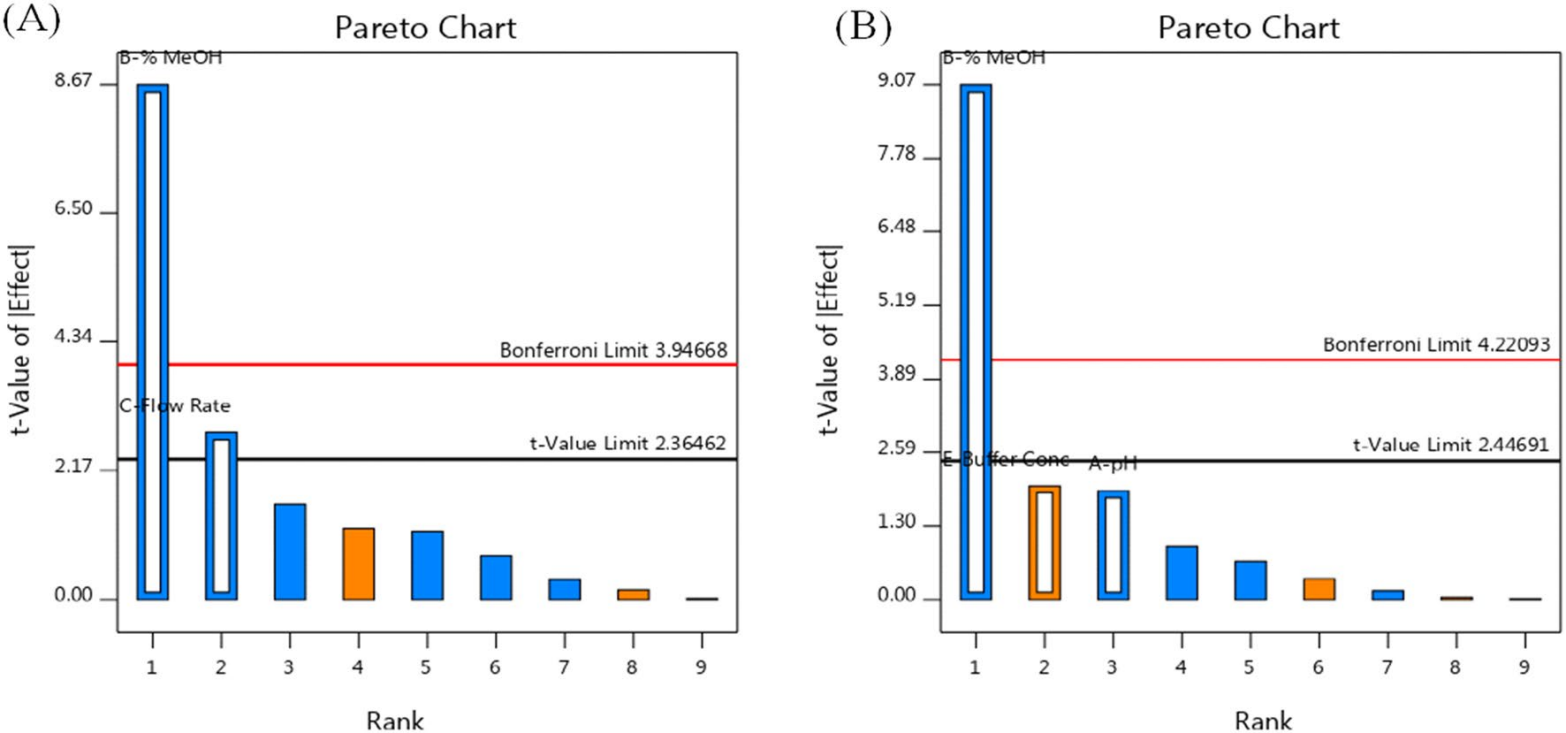
Pareto chart showing factors effect on: (**A**) retention time (VAL) and (**B**) resolution between XIP and valsartan (VAL)

Analysis of variance (ANOVA) for the studied factors is given in Table 5. The results indicated that only flow rate and % MeOH were the significant variables. Pareto charts, presented in Fig. 3, showed that flow rate had a significant effect only on the retention time (VAL), while % MeOH was a critical variable for both responses.

**Table 5:** ANOVA results of the fractional factorial design (insignificant interaction effects were excluded)

Item	Retention time (VAL) (min)		Resolution	
	F	p-value	F	p-value
A: pH	–	–	3.66	0.1041
B: % MeOH	75.19	< 0.0001	82.35	0.0001
C: Flow rate	7.91	0.0260	–	–
D: Detection wavelength	–	–	–	–
E: Buffer conc	–	–	4.02	0.0919
Adjusted R^2^	0.9132		0.9110

#### Optimization with CCD

The results calculated by ANOVA of the significant factors are mentioned in detail in Table 6. Results confirmed the previous factors effects obtained by screening ANOVA. In addition, quadratic effects on retention time (VAL) were observed, while 2FI model was suggested for resolution.

**Table 6:** Regression coefficients of polynomial equation along with p-value of ANOVA of central composite design

Item	Retention time (VAL), min		Resolution^1	
	Coefficient	p-value^a^	Coefficient	p-value^a^
Intercept	5.84		6.19077	
A—% MeOH	−1.09822	**< 0.0001**	−1.44686	** < 0.0001**
B—Flow rate	−0.519041	**< 0.0001**	−0.0768198	**0.0015**
AB	0.0925	0.0837	0.1	0.0025
A^2^	0.17625	**0.0015**	–	–
B^2^	0.04875	0.2040	–	–
Model	Quadratic	**< 0.0001**	2FI	**< 0.0001**
Adjusted R^2^	0.9917		0.9983	

^a^bold p-values indicates significant effect

**Fig. 4:**
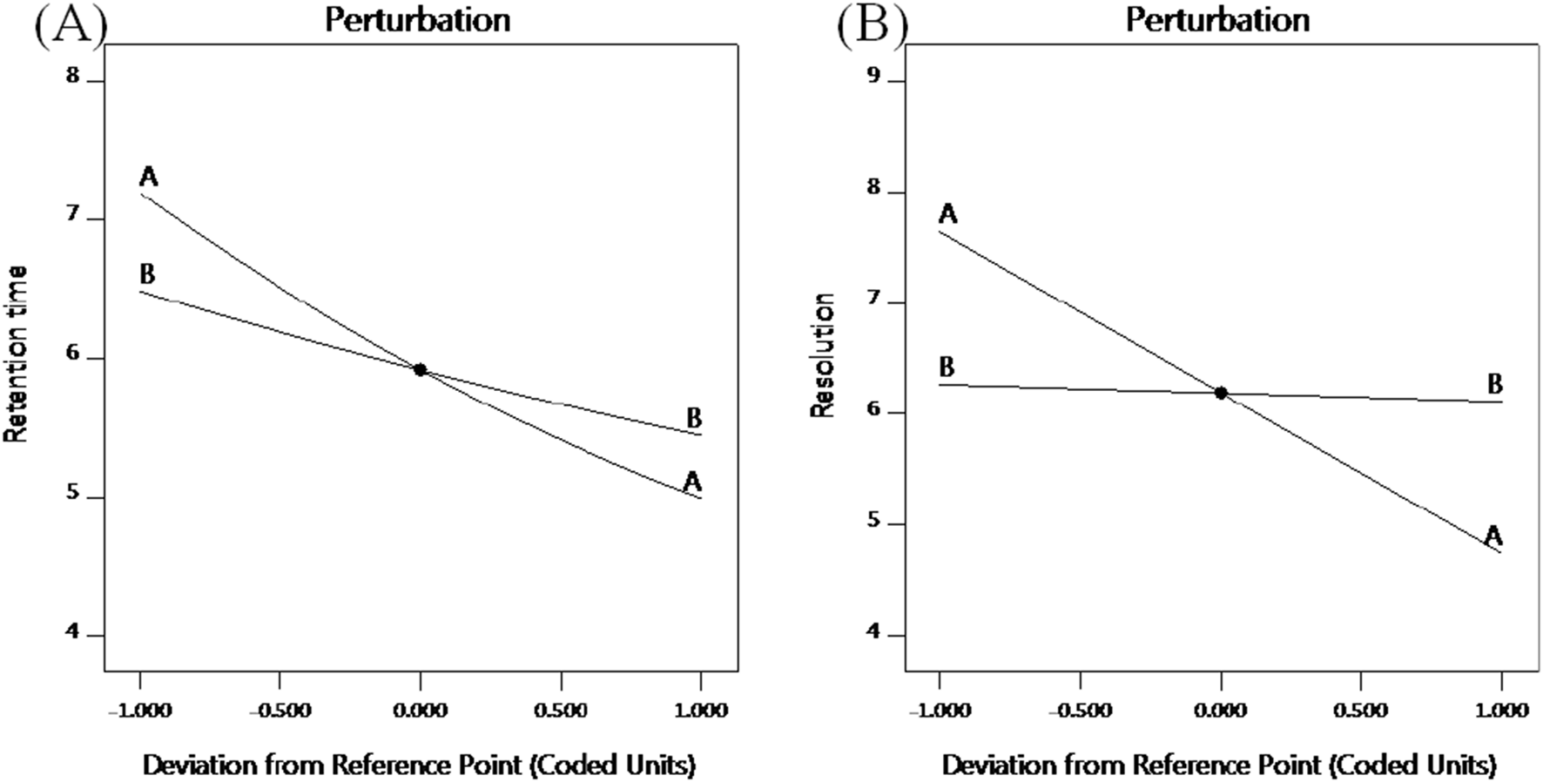
Perturbation plot for effect of factors on: (**A**) retention time (VAL) and (**B**) resolution, where line (**A**) is % MeOH and line (**B**) is flow rate

**Fig. 5:**
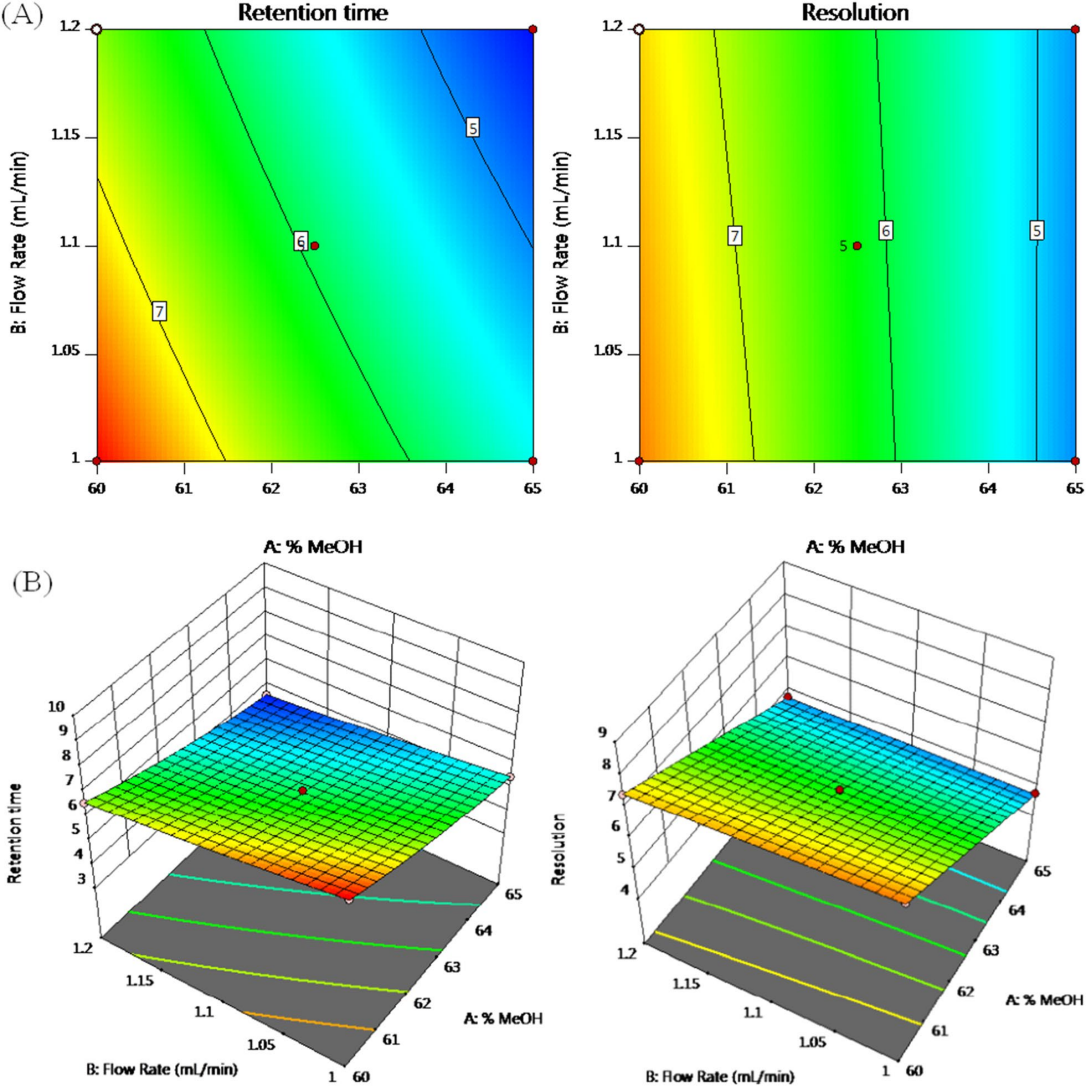
Contour (**A**) and 3D (**B**) plots showing the interaction effect of the % MeOH and flow rate on retention time (VAL) and resolution

Perturbation figure shows that % MeOH and flow rate had the most significant negative effect on retention time (VAL), Fig. 4A; increasing the variables was followed by a decrease in the response. The quadratic effect of % MeOH (factor A) is confirmed by the curvature of line A. On the other hand, % MeOH showed a similar effect on resolution (Fig. 4B). Contour and 3D plots (Fig. 5) show the interaction effect of the critical factors on retention time (VAL), and on resolution. Numerical optimization solution suggested those following optimal conditions: 64.5% MeOH, and a 1.2 mL/min flow rate. These optimal conditions have a desirability function of 0.716.

**Fig. 6:**
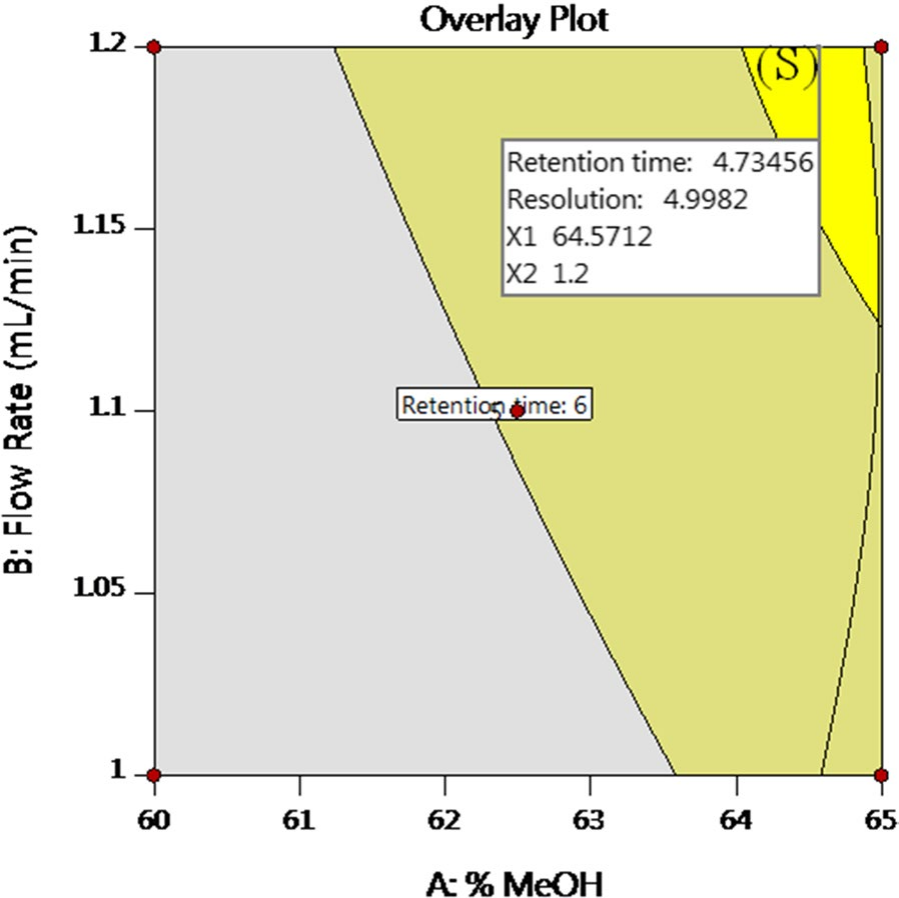
Overlay plot showing the sweet spot (S) where the desired responses met

The overlay plot represents the best desirable requirements of factors, responses and POE which are met in the sweet spot (S) as depicted in Fig. 6. Then, the variables optimum ranges were determined using the overlay contour plots as: % MeOH 63.95–64.99% and flow rate 1.12–1.2 mL/min. These ranges are representing the design space and confirm method robustness.

The responses predicted means and their POE were reported within the low and high PI 95%, thus confirming predictability of the model. Additionally, the percentage prediction error was equal to −0.718 and 0.474 for retention time (VAL) and resolution, respectively (predicted retention time (VAL) = 4.734 and resolution = 4.998).

The following quadratic equation shows the relation between the significant factors and the selected responses (y):

Y = b_0_ + b_1_A + b_2_B + b_3_AB_+_ b_4_A^2^ where b_0_ is the intercept, b_1_–b_5_ represents the regression coefficients of quadratic polynomials and 2FI for both responses (Table 6).

### Method validation

The method validation was performed according to food and drug administration [43–45].

#### Linearity

**Fig. 7:**
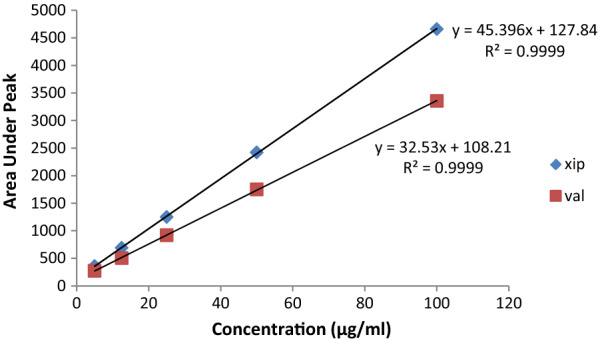
Calibration curves for authentic mixture of XIP and valsartan VAL using the proposed HPLC method

Five different concentrations of the drug mixture were specified for linearity studies in the range of 5–100 µg/mL for both drugs (Table 7). Linear regression equations of XIP and VAL were found to be y = 45.396x + 127.84 and y = 32.53x + 108.21, respectively and the regression coefficient values (r) were calculated to be 0.9999 for both drugs indicating a high degree of linearity (Fig. 7).

**Table 7:** Analytical merits for determination of XIP and VAL in pure samples using the proposed HPLC method

	XIP				VAL			
	Conc. taken (µg/mL)	Conc. found (µg/mL)	Recovery %	Accuracy (RE %)	Conc. taken (µg/mL)	Conc. found (µg/mL)	Recovery%	Accuracy (RE %)
	5	5.07	101.4	1.4	5	5.06	101.31	1.31
	12.50	12.38	99.06	−0.93	12.50	12.19	97.58	−2.41
	25	24.71	98.87	−1.12	25	24.95	99.82	−0.17
	50	50.49	100.98	0.98	50	50.47	100.94	0.94
	100	99.83	99.83	−0.16	100	99.8	99.8	−0.19
Mean			100.03	0.03			99.89	−0.1
SD			1.12				1.45	
CV (%)			1.13				1.46	
SE			0.5				0.65	
Variance			1.27				2.11	
Slope			45.93				32.52	
LOD (µg/mL)m			0.075				0.134	
LOQ (µg/mL)m			0.248				0.448

#### Accuracy

The accuracy of the proposed method was indicated by % recovery of the two different concentrations of XIP and VAL in human plasma.

#### precision

The method precision was evaluated in terms to intra-day and inter-day precision using the validation QC samples at concentrations of 12.50, 25 and 50 µg/ml. Intra-day precision was evaluated depending on standard deviation (SD) & coefficient of variation (CV%) where three replicates using the same solution of pure drugs were used. The SD values (ranged from 0.12 to 0.37) and CV% values (ranged from 0.12 to 0.38) indicated that the method is highly precise. Also, for inter-day reproducibility, SD & CV% values were in the acceptable range of 0.06–0.52 and 0.06–0.53, respectively (Table 8). These results show that the proposed method has an adequate precision in simultaneous determination of both drugs in either pharmaceutical or biological samples.

**Table 8:** Intra- and inter-day precision and stability results of XIP and VAL QC samples samples

	Drugs	Concentrations (µg/mL)	Mean^a^ ± SD	CV (%)
Intra-day runs (n = 3)	XIP	50	100.98 ± 0.12	0.12
		25	98.91 ± 0.19	0.19
		12.5	98.97 ± 0.37	0.38
	VAL	50	100.53 ± 0.13	0.1
		25	99.57 ± 0.16	0.17
		12.5	98.06 ± 0.34	0.35
Inter-day runs (n = 3)	XIP	50	100.8 ± 0.06	0.06
		25	98.92 ± 0.31	0.32
		12.5	99.15 ± 0.37	0.38
	VAL	50	100.75 ± 0.08	0.08
		25	99.84 ± 0.3	0.3
		12.5	98.19 ± 0.52	0.53
3 Freeze–thaw cycles at − 20 °C (n plasma = 3)	XIP	20	93.02 ± 0.38	0.41
		15	95.62 ± 0.83	0.87
		5	98.31 ± 0.62	0.63
	VAL	20	87.72 ± 0.21	0.24
		15	85 ± 0.86	1.02
		5	99.77 ± 2.17	2.17

^a^Average of three determinations

#### Selectivity and specificity

The method selectivity was checked by injecting XIP and VAL solutions separately into the column where 2 sharp peaks were eluted at retention times of 3.4, and 4.6 min, respectively, and these peaks were not monitored for the blank solution.

#### Limits of detection and limits of quantification

For estimating the limits of detection and quantification, the method reported by Bhaskaran et al. [46] was used based on equations: LOD = 3.3 σ/s and LOQ = 10 σ/s, where, σ is SD of y-intercepts of the regression line and s is the slope of the calibration line. LODs were reported to be 0.075 and 0.134, while LOQs were calculated to be 0.248 and 0.448 µg/mL for both XIP and VAL, respectively (Table 7) showing that the proposed method is highly sensitive and being applicable for future bioequivalence studies where it is mandatory to detect small drug concentrations in plasma.

#### Stability

Stability and precision studies were also conducted through application of plasma freeze–thaw cycles at −20 °C (over 3 days) using validation samples (5, 15 and 20 µg/mL of XIP and VAL) in plasma (Table 8). The recoveries for XIP and VAL were reported to be 93.09% and 89.17%, respectively as presented in Table 9.

**Table 9:** Result of analysis of proposed method in human plasma

Parameters	XIP				VAL			
	Taken µg/mL	Found µg/mL	Recovery^a^ %	Accuracy (RE %)	Taken µg/mL	Found µg/mL	Recovery^a^ %	Accuracy (RE %)
	20	18.77	93.85	−6.14	20	17.73	88.65	−11.34
	15	14.58	97.24	−2.75	15	12.96	86.44	−13.55
	5	5.00	100.07	0.07	5	4.97	99.47	−0.52
	2.5	2.03	81.2	−18.79	2.5	2.05	82.12	−17.87
Mean			93.09	−6.9			89.17	−10.82
± SD			8.32				7.38	
± CV (%)			8.94				8.27	
± SE			3.72				3.3	
Variance			69.3				54.47	

^a^Average of three determinations

### Analysis of human plasma

XIP is well absorbed with maximum observed plasma concentration (C_max_) occurring 1 h of oral doses. C_max_ after oral administration of 20 mg is 3 μg/mL [47]. VAL is rapidly absorbed after administration of tablets and oral solution with bioavailability of 23% and 39%, respectively. It is not significantly metabolized, so it is excreted mainly as unchanged form via the bile [7]. Following a single oral dose of 80 mg, C_max_ is approximately 3.128 ng/mL with a t_max_ of 1.5 h for oral solution [48].

The proposed method was adopted for determination of XIP and VAL in human plasma by applying protein precipitation procedure. XIP and VAL retention times in plasma samples and the other system suitability parameters were pretty similar to those values in pure ones (Table 4). Also, the plasma chromatogram (Fig. 2A) confirms the method specificity in clinical studies as the plasma peak is not interfering with both XIP and VAL peaks.

### Comparison with the reported method

Analytical parameters of the developed method were compared with some of the previously reported ones for estimation of VAL. The comparison presented in Table 10 shows that the developed procedure has the shortest run time. In addition, none the reported method used CCD for method optimization; CCD is superior to full factorial design (FFD) that is not generally advised in optimization procedures because of its incapability of examining quadratic models. Therefore, FFD can be used only for mapping linear relationships while CCD help obtaining more reliable models [49]. Moreover, the rotatable CCD applied in this study is better than FFD and other CCD; it uses five variable levels and consequently, can provide more accurate results. In term of greenness, the proposed mobile phase is the most eco-friendly. Therefore, this study could be considered as a promising would show a better performance. In addition, statistical analysis showed no significant difference between the two methods.

**Table 10:** Comparison of the proposed and reported methods for determination of VAL

Item	Proposed method	Reported method [17]	Reported method [18]	Reported method [19]
Technique	HPLC–UV	HPLC–UV	HPLC–UV	HPLC–UV
Matrix	Human plasma	Nano-formulation	Rabbit Plasma	Nano-formulation
Optimization strategy	Central Composite Design	Full factorial design	Full factorial design	One factor at a time
Mobile phase	Methanol: 0.05 M phosphate buffer, pH 3 (64.5:35.5, v/v)	Acetonitrile: 20 mM ammonium formate, pH 3 (43:57, v/v)	Acetonitrile: 20 mM ammonium formate (42:58 v/v)	Acetonitrile: 10 M phosphate buffer, pH 3.6 (60:40, v/v)
Analytes	XIP and VAL	VAL	VAL	VAL
LOD (ng/mL)	134	4.833	22.000	6.000
LOQ (ng/mL)	448	44.95	66.67	25
Retention time (min)	4.34	10.177	11.394	2.91
% Recovery ± SD	89.17 ± 7.38		94.81 ± 9.80	
n	4		3	
V	VAL: 54.47		96.13	
t	0.835 (2.571)^a^			
F	1.765 (9.53)^b^			

^a,b^Tabulated t values and F ratios at p = 0.05

## Conclusion

QbD strategy was adopted to develop a robust and an efficient RP-HPLC method for simultaneous estimation of xipamide and valsartan mixture in human plasma. Multivariate regression analysis was successfully carried out to study the main effects of 4 factors on both column efficiency and resolution. CCD was carried out to optimize of chromatographic conditions through studying the interaction and quadratic effects of significant factors on the two selected responses. The models which were used for screening and optimization steps were highly significant and confirmed the method predictability. The method is very simple, accurate, robust, and can be applied successfully to the analysis of XIP and VAL in human plasma with a high degree of selectivity.

## Data Availability

All data generated or analyzed during this study are included in this published article.
